# Influence of Prebiotic Activity of *Agave salmiana* Fructans on Mucus Production and Morphology Changes in Colonic Epithelium Cell of Healthy Wistar Rats

**DOI:** 10.3389/fpls.2021.717460

**Published:** 2021-12-13

**Authors:** Amneris Iraida Castillo Andrade, Erika García Chávez, Cecilia Rivera Bautista, Cuauhtemoc Oros Ovalle, Miguel Angel Ruiz Cabrera, Alicia Grajales Lagunes

**Affiliations:** ^1^Facultad de Ciencias Químicas, Universidad Autónoma de San Luis Potosí, San Luis Potosí, Mexico; ^2^Instituto de Investigación de Zonas Desérticas, Universidad Autónoma de San Luis Potosí, San Luis Potosí, Mexico; ^3^Departamento de Patología, Hospital Central Dr. Ignacio Morones Prieto, San Luis Potosí, Mexico

**Keywords:** *Agave salmiana*, fructans, epithelium, mucus, prebiotic activity

## Abstract

The beneficial health of evaluating prebiotic effect by the consumption of *Agave salmiana* fructans (*A. salmiana* fructans) was assessed in the epithelium of the cecum and proximal colon of Wistar rats fed at different doses for 35 days with regards to mucus production, morphological cell changes, and the serum concentration of tumor necrosis factor-α (TNF-α). Results showed a significant increase in mucus-secreting cells (*P* < 0.05) and a normal structure with preserved crypts, without morphological damage to colonic cells for a dose of 12.5% (w/w) with respect to the control and the other doses evaluated. The concentration of pro-inflammatory cytokine TNF-α was decreased significantly (*P* < 0.05) in the groups with doses of 10 and 12.5% (w/w) at 7 and 35 days, respectively. This effect was positively correlated with the reduction of inflammation in epithelial cells. This study provides direct evidence of the effects of the *A. salmiana* fructans on the colonic epithelium, demonstrating that a diet supplemented with 12.5% of fructans for 35 days exerts health benefits through the strengthening of the mucosa layer, which favors the adherence of the bacterial population and suppresses inflammation.

## Introduction

*Agave* fructans (*A.* fructans) represent a category of branched natural compounds. Furthermore, they have a complex structure with a fructose-fructose glycosidic ß (2-1) and ß (2-6) bond, with one terminal glucose unit. Because of the branched structure and type of linkage β of these molecules, they have been used as a prebiotic ingredient. Prebiotic is a substrate selectively utilized by commensal host microbiota, which confers a health benefit (Gibson et al., 2017). Previous studies have reported that *A. salmiana* fructans exert beneficial physio-metabolic effects on the host, commensal bacteria growth, and metabolic activity, particularly, on short-chain fatty acids (SCFAs) as products of microbiota fermentation in the colon. Therefore, its consumption could decrease the risk of gastrointestinal diseases (Moreno-Vilet et al., 2014; Jasso-Padilla et al., 2016; Martinez-Gutierrez et al., 2017; Castillo Andrade et al., 2018, 2019). The effect of *A. salmiana* fructans on mucus production and the morphology of the epithelial cells, however, has not been reported. The gut epithelium constitutes a physical barrier that regulates the transcellular and paracellular transit of exogenous substances and impairs the entry of most paracellular cells. This barrier is strengthened by the mucus layer integrated by glycoproteins (mucins), the synthesis of antimicrobial peptides, and other secretions (bile, organic acids, and enzymes; Laparra and Sanz, 2010). The dietary intake of prebiotics promotes mucus secretion, thus, improving colonic barrier function (Brownlee et al., 2003). In addition, it is known that the endogenous probiotic bacteria of the colon produce SCFAs through the fermentation of fructans, which has a positive impact on the regulation of the gut homeostasis, while subsequently suppressing intestinal inflammation. Butyrate provides 70% of the colonocytes metabolic needs, by controlling normal colonic mucosal homeostasis through its proliferative and apoptotic properties in healthy and transformed tissues (Daly et al., 2005). Beyond its nutritional impact on colonic epithelial cells, butyrate regulates fluid and electrolyte uptake, influencing epithelial cell cytokines, and enhancing barrier function (Canani et al., 2012; Schaafsma and Slavin, 2015). Several authors have shown an increase in SCFA production when fructans are consumed (Van de Wiele et al., 2004; Paturi et al., 2015; Paßlack et al., 2015: Ramnani et al., 2015; Castillo Andrade et al., 2019). The prebiotic effect of fructans on epithelial cells and the production of SCFAs could be dependent on the dose and branched prebiotic. The excessive SCFAs and higher endotoxin production derived from gram-negative bacteria induce acute ruminal acidosis leading to severe damage to rumen epithelium (Tao et al., 2014). Castillo Andrade et al. (2019) showed that rats fed with high doses (15 and 20%) of *A. salmiana* fructans experienced a loss of cells, a disruption and disappearance of the mucous layer, and the presence of hemorrhages and inflammatory infiltrates. In addition, some proof has been found that changes in gut epithelium produce a disturbed balance between pro-inflammatory and anti-inflammatory cytokines, with increased levels of the pro-inflammatory cytokine interleukin-1 (IL-1), interleukin-6 (IL-6), interleukin-8 (IL- 8), and tumor necrosis factor-α (TNF-α) (Rogler and Andus, 1998). Therefore, this study aimed to evaluate the influence of *A. salmiana* fructans consumption at different doses (5, 10, 12.5, 15, and 20%) on mucus production and the morphological changes of the cecum and proximal colon of Wistar rats through histological analyses and scanning electron microscopy (SEM). Additionally, the quantification of TNF-α was also performed as a direct measurement of suppressing inflammation.

## Materials and Methods

### Attainment and Characterization of the *Agave salmiana* Fructans Powder

Six heads of *A. salmiana* Otto ex Salm–Dyck were collected in the community of Charcas, in the state of San Luis Potosi, Mexico, taking into account the complete physiological ripeness features of the plant to ensure high fructan content according to Aguirre-Rivera et al. (2001). The heads with plant number 034802 were identified by the Instituto de Zonas Desérticas de la Universidad Autónoma de San Luis Potosí. For the extraction of fructans, the juice extracted from the stem of *A. salmiana* was filtered in a stainless steel press filter (Shriv 405 type) to eliminate all fibers, and later, it was subjected to 80°C for 30 min in a water bath with continuous agitation to inactivate the saponins. The juice was then concentrated at 42 ± 1°C by using cooking and vacuum impregnation equipment (J.P. Selecta Gastrovac, Barcelona, Spain) until the extract had a consistent density of approximately 50 Brix. The concentrates were subsequently dried (to approximately 95% dry matter) in a forced-air oven (SHEL LAB FX14, United States) at 53 ± 2°C. With this process, an *A. salmiana* fructans purity of 88% was obtained (Castillo Andrade et al., 2019).

### Animals and Diet

The animals were handled according to the specifications for care and management described in the official Mexican standard NOM-062-ZOO-1999. The experimental procedure was approved by the local University Ethics Committee for Animal Research under registration number CONBIOÉTICA24CEI00820131212. Eight-week-old male Wistar rats (*n* = 48) were housed individually in acrylic cages containing pressed wood chips as bedding. The room housing the rats was maintained at a temperature of 22 ± 1°C and 50 ± 5% humidity, with air exchanged 12 times/h, and a 12-h light/dark cycle. After 7 days of acclimation, the animals were randomly distributed into six groups (*n* = 8/group). The control group received a standard diet (Lab Diet 5001, Prolab RMH 2500) without fructans. The other groups received a standard diet supplemented with different concentrations (w/w) of fructans (5, 10, 12.5, 15, and 20%). The pellets were prepared according to Castillo Andrade et al. (2018). All rat groups were given *ad libitum* access to water and 35 g/day of pellets with the corresponding dose for 35 days. The daily quantity of food (35 g/day) was according to the feeding directions suggested by the Prolab RMH 2500 diet. The animals were fed once a day for 35 days. Every 7 days, blood samples were taken by puncturing the caudal vein through the MARPER drip technique (1.2 ml blood sample) prior to fasting for 12 h (Martin-Pérez, 2000). After 35 days of treatment, the rats were euthanized through a pentobarbital sodium overdose (80 mg/kg), and blood samples were collected by heart puncture. Serum was separated by centrifuging the blood at 3,000 rpm for 10 min and stored at –20°C for quantification of TNF-α. The cecum and colon organs were carefully removed under sterile conditions. The organs were immediately snap-frozen in liquid nitrogen and stored at –80°C for subsequent analysis. All animals were observed throughout the experiment for mortality and clinical signs of morbidity.

### Histological Analyses of the Cecum and Proximal Colon

Cecum and proximal colon organs were thawed at room temperature, cut transversely in sections of approximately 1 cm, and fixed in 10% buffered formalin. Tissue fragments were embedded in paraffin, and two stained types were done. A portion of the tissues was stained with hematoxylin and eosin to quantify the mucus production and variations of cell nuclei length. To obtain the average of cell nuclei length, 10 individual nuclei were measured using a micrometric ruler of the software cellSens. Another part was stained with Masson staining to determine general features, connective tissues, and collagen fibers. The images of every tissue were obtained at 10 × and 40 × magnification using a digital camera (an Olympus DP27, Tokyo, Japan) mounted on a CX31 microscope (Olympus, Tokyo, Japan) connected to a computer. Once captured, the images were processed using cellSens imaging software version 1.16 of Olympus.

### Scanning Electron Microscopy of the Cecum and Proximal Colon

Scanning electron microscopy was performed in the tissues of rats that consumed 0, 12.5, and 15% of the dose to detect changes in mucosa ultrastructure associated with the consumption of fructans. The cecum and proximal colon were thawed at room temperature and cut longitudinally along the minor curvature. The tissues were washed with phosphate-buffered saline (PBS) a pH 7.3 to remove all fecal matter and endogenous bacteria according to the technique described by Barnali and Ghosh (2014) with modifications. The tissue sections were fixed in 2.5% glutaraldehyde for 2 h at room temperature. Immediately following, the tissues were dehydrated in graded ethanol as follows: 50% (30 min), 70% (45 min), and 90% (60 min). They were then kept in absolute ethanol overnight. Transversal sections of approximately 10 μm were obtained with a microtome. Finally, the tissues were coated with palladium gold in a Sputter coater for 5 min and placed under a scanning electron microscope (TOPCON SM 510) for observation and subsequent photography.

### Quantification of Pro-inflammatory Cytokine TNF-α

Tumor necrosis factor-α concentration in serum was quantified by enzyme-linked immunosorbent assay (ELISA) at 7 and 35 days after treatment by using the commercially available rat TNF-α ELISA kit (900-K73 PeproTech, Cranbury, NJ, United States). The procedure was performed following the instructions of manufacturer. Optical densities were measured with an ELISA reader at a 450-nm wavelength. The amount of TNF-α was calculated using the standard curve generated from recombinant proteins for TNF-α [*y* = 0.691^∗^ln(x) – 1.222, R^2^ = 0.976].

## Statistical Analysis

Tumor necrosis factor-α concentration and nucleus size data were analyzed through an ANOVA with a Tukey test to detect differences between doses. A probability value of *P* < 0.05 was considered significant.

## Results

### Histological Analyses of the Cecum and Proximal Colon

**FIGURE 1:**
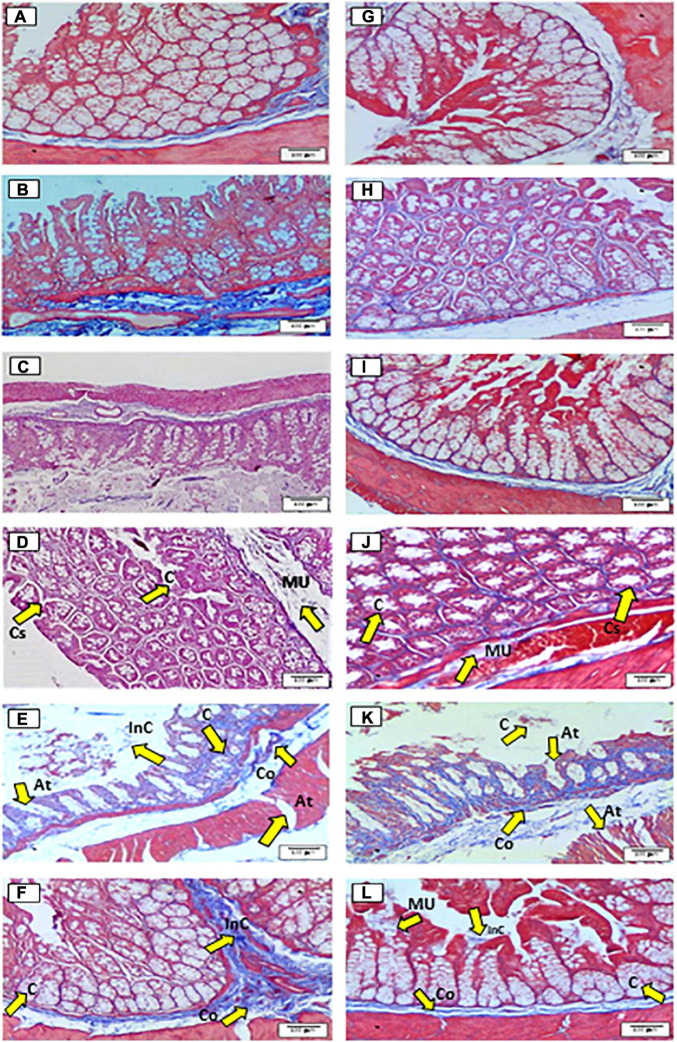
Histological effects of the diets on the general structure of the epithelium of the cecum **(A–F)** and proximal colon **(G–L)** in rats fed for 35 days with different doses of *Agave salmiana* fructans. In addition, panels **(A,G)** are the control of the cecum and proximal colon, respectively, and panels **(B,H)** are the doses of 5%, with panels **(C,I)** of 10%, panels **(D,J)** of 12.5%, panels **(E,K)** of 15%, and panels **(F,L)** of 20%. At, atrophy; C-cells, co-collagen; Cs, cellular spaces; InC, inflammatory cell infiltrates; MU, mucosa.

Masson staining showed the general features of the cecum (Figures 1A–F) and proximal colon (Figures 1G–L). The results of histological analyses for the doses of 5–12.5% (Figures 1B–D,H–J) showed a normal structure of the cecum and proximal colon tissues with good morphology of intestinal glands, preserved crypts with well-defined spaces between crypts, a normal coating surface, and loose connective tissue, which included blood vessels and submucosal nerve plexuses compared with control and the other doses. Particularly, the mucosa layer of the cecum and proximal colon for the 12.5% dose (Figures 1D,J) showed a heterogeneous surface with indices of cellular regeneration on the surface of crypts and a hyper-secretory effect of mucus with an increase in the number of goblet cells surrounded by a network of muscle fibers.

The 15% dose (Figures 1E,K) showed severe damage in the structure, such as the uniform dilation of the mucosal wall, the disorganization of aberrant crypts with irregular sizes in the cecum, and proximal colon. In addition, a decrease in the number of mucus-secreting cells and inflammatory cell infiltrates was also observed, and patches of atrophy and dense deposits of collagen were also observed, which indicated severe fibrosis. Concerning the 20% dose (Figures 1F,L), the epithelium structure was not affected uniformly as was observed in the dose of 15%. It is important to note that in the 20% dose, signs of cellular repair and mucosal scarring were found, whereby the tissues showed a normal glandular density.

**FIGURE 2:**
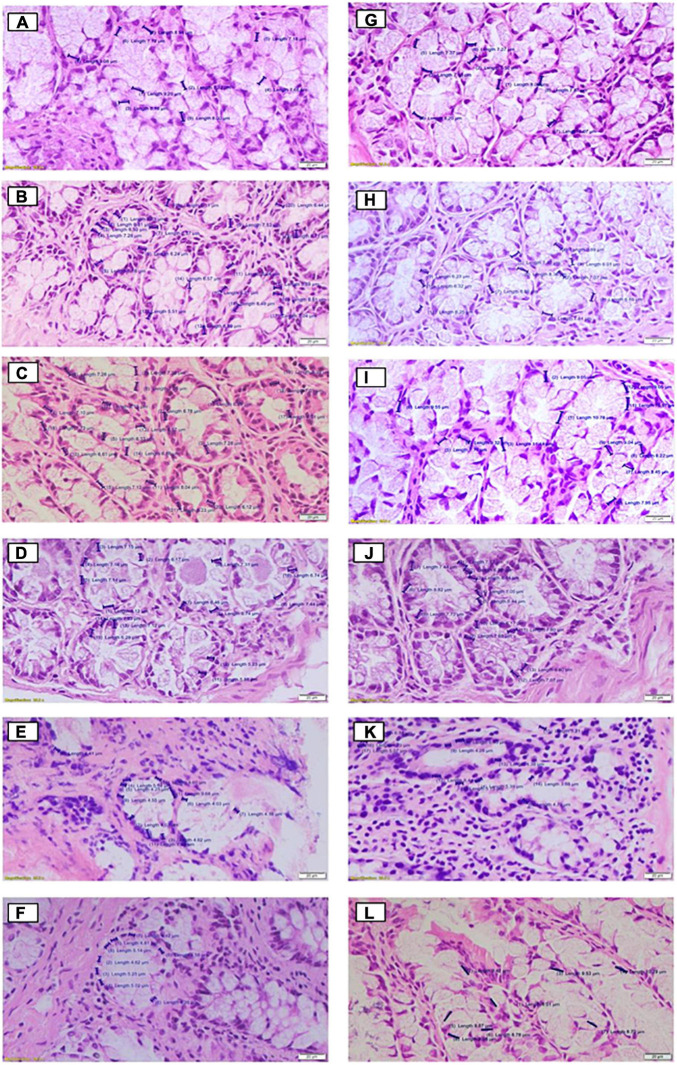
Histological effects of the diets on cell nucleus size in the cecum **(A–F)** and proximal colon **(G–L)** of rats fed for 35 days with different doses of *Agave salmiana* fructans, panels **(A,G)** are the control of the cecum and proximal colon, respectively, and panels **(B,H)** are the doses of 5%, with panels **(C,I)** of 10%, panels **(D,J)** of 12.5%, panels **(E,K)** of 15%, and panels **(F,L)** of 20%.

**FIGURE 3:**
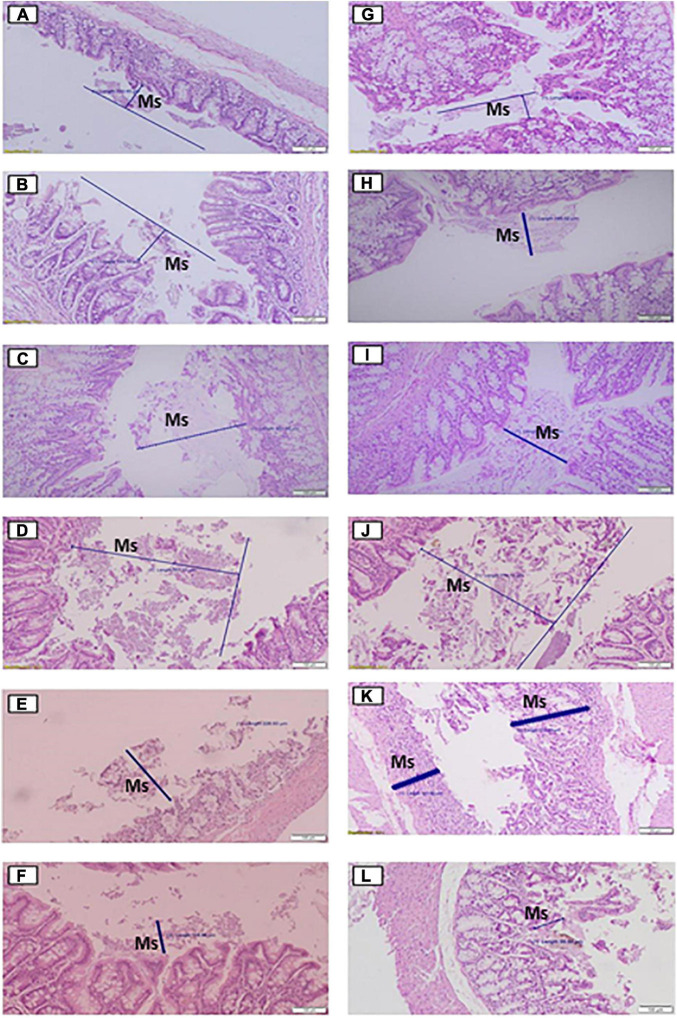
Histological effects of the diets on mucus production in the cecum **(A–F)** and proximal colon **(G–L)** of rats fed for 35 days with different doses of *Agave salmiana* fructans. In addition, panels **(A,G)** are the control of the cecum and proximal colon, respectively, and panels **(B,H)** are the doses of 5%, with panels **(C,I)** of 10%, panels **(D,J)** of 12.5%, panels **(E,K)** of 15%, and panels **(F,L)** of 20%. Ms, mucus.

In relation to the nucleus size and the quantification of mucus production, the data are summarized in Table 1, and the respective images are shown in Figures 2A–L, 3A–L, respectively, for the cecum and proximal colon. The supplementation of *A. salmiana* fructans at 5, 10, and 12.5% showed no significant effect (*P* > 0.05) in the nucleus length with respect to the control group (Figures 2A–D,G–J). A significant decrease (*P* < 0.05) of the nucleus length was observed in the cecum for the doses of 15 and 20% (Figures 2E,F). In the proximal colon, only the 15% dose (Figure 2K) showed a significant decrease (*P* < 0.05) of the nucleus length, as was observed in the cecum. In addition, the doses of 15 and 20% presented evident cytotoxicity changes, through nuclear disorganization with the migration of the nuclei toward the mucosa, the presence of nuclear polymorphism, and a decrease in the size of vacuoles in the cecum and proximal colon.

**TABLE 1:** Nucleus length and mucus size in the cecum and proximal colon.

**Dose (%)**	**Nucleus length (mm) Nucleos**		**Mucus size (mm)**	
	**Cecum**	**Proximal colon**	**Cecum**	**Proximal colon**
0	7.58 ± 0.33^a^	7.75 ± 0.41^a^	103.45	99.09
5	7.11 ± 0.29^a^	7.47 ± 0.64^a^	144.10	165.02
10	7.20 ± 0.11^a^	8.29 ± 0.49^a^	361.37	240.03
12.5	7.22 ± 0.13^a^	7.95 ± 0.49^a^	551.07	516.15
15	4.44 ± 0.21^b^	4.65 ± 0.29^b^	226.93	259.28
20	4.54 ± 0.21^b^	8.80 ± 0.52^a^	126.86	99.56

*The results are the mean ± SD.*

*Groups with the same letter showed no statistically significant difference, whereas groups with different letter designation different (*P* < 0.05).*

The consumption of *A. salmiana* fructans in doses of 5–12.5% showed an increase in the mucus production in both tissues (Figures 3A–D,G–J), with five times more for the dose of 12.5% compared with the control. The doses of 15 and 20% showed a decrease in mucus production in both tissues (Figures 3E,F,K,L) compared with the dose of 12.5%. The mucus produced in the dose of 20%, however, was similar to the control but less than the dose of 15% in the cecum and proximal colon. In addition, for the dose of 20% signs of scarring, macrophages and CD4 T lymphocytes were observed in mucosa, submucosa sections, abnormal mitosis, loss of mucosa contours, and the loss of epithelium because of the extensive mucosa damage from these doses.

### Scanning Electron Microscopy of the Cecum and Proximal Colon

**FIGURE 4:**
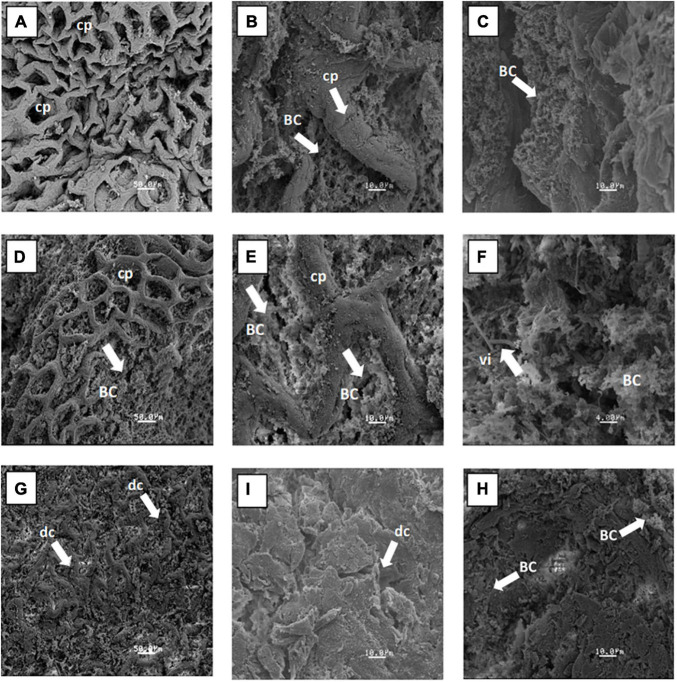
Scanning electron micrograph images of the general structure of epithelium and bacteria adhering to the mucus layer of rats fed with fructans at the following doses: dose 0% **(A,B)** cecum and **(C)** proximal colon; 12.5% **(D,E)** cecum and **(F)** proximal colon; and 15% **(G,H)** cecum, and **(I)** proximal colon. BC, bacterial colonization; cp, crypts preserved; dc, disruption of crypts; vi, villus.

The ultrastructure of the cecum and proximal colon using a scanning electron microscope is shown in Figures 4A–I for the doses of 0, 12.5, and 15%. In these images, the increase in the bacterial colonization inside of crypts was observed, and areas completely full of bacillus groups can be seen as compared with the control group. In addition, the dose of 12.5% showed normal structures with preserved crypts and with an increase in the thickness of the mucosa layer, as was observed previously in the histological analysis. The ultrastructure of the proximal colon for the 12.5% dose showed an increase in the length of numerous cylindrical villi and an increase in the bacterial population adhering to the rat colon epithelium. The 15% dose caused evident atrophy in the cecum and proximal colon, which was shown by severe disruption of the crypts of the cecum and a decrease of the bacterial population in both tissues.

### Quantification of Pro-inflammatory Cytokine TNF-α

**FIGURE 5:**
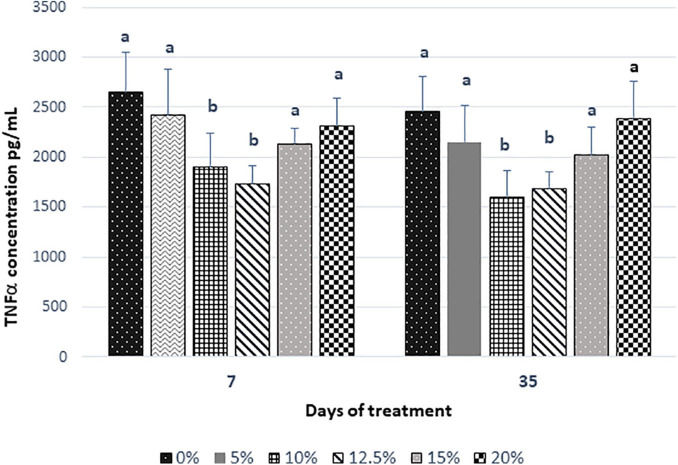
The serum concentration of TNF-α of rats fed for 35 days with *Agave salmiana* fructans. TNF-α, tumor necrosis factor-α.

The results of the pro-inflammatory cytokine TNF-α are shown in Figure 5. In the groups of rats that received fructans, there was a lower level of TNF-α post-treatment in the serum as compared with the control group. Our results showed that the level of pro-inflammatory cytokine TNF-α was significantly (*P* < 0.05) reduced in the groups with the doses of 10 and 12.5% after 7 days post-treatment. At 35 days of treatment, a slight decrease was observed in the control group and all groups that consumed fructans except for the doses of 20% that remained constant. The percentage of decrease at 7 days post-treatment for the doses of 5, 10, 12.5, 15, and 20% with respect to the control was 9, 28, 34, 20, and 13%, respectively. If we consider the TNF-α values at 7 and 35 days, we can obtain the percentage of decrease between 7 and 35 days for all doses including the control group. The percentage of the decrease of TNF-α levels at 35 days for the control and the doses of 0, 5, 10, 12.5, 15, and 20% were 7, 11, 16, 4, 5, and 0%, which were lower than the levels obtained for 7 days after treatment.

## Discussion

According to the literature, the goblet cells secrete mucin glycoproteins that form a thick mucosa layer overlying the intestinal epithelium that provides the first line of defense in innate immunity (Paturi et al., 2015). This current study showed that the consumption of *A. salmiana* fructans keeps the cecum and proximal colon cells intact in the doses of 0, 5, 10, and 12.5%. Maintaining goblet cells structure ensures the integrity of the mucosa layer, which could help prevent the inflammation of the colonic epithelium. It has been reported that the peculiar biological architecture, appearance, and glandular formation of the gastrointestinal mucosa permit the distinction between harmful pathogens and protective symbiotic microorganisms, generating a strong effect for maintaining the intestinal microbiota under control by a “constitutive low-grade physiological inflammation” (Biagi et al., 2012). Additionally, it was demonstrated *in vitro* that the inulin-type fructans promote a decreased rate of aberrant crypt foci in rats, which are the earliest changes that lead to colon cancer. This decrease was related to a stable butyrate-producing colonic ecosystem, thus adding to the line of evidence of potential effects of fructans for reducing the risks of developing colon cancer (Perrin et al., 2001). In a previous study, we reported that the dose of 12.5% produced a high butyrate concentration in cecal content because of the fermentation of *A. salmiana* fructans (Castillo Andrade et al., 2019). Therefore, the increase in the mucus production and the increase in villi length observed in the 12.5% dose in colonic epithelium may be attributed to the SCFAs produced. The SCFAs are well known for their health-promoting effects, such as their trophic effect on the intestinal epithelium and their stimulatory effect on sodium, chloride, potassium, and water absorption from the colonic lumen (Bedford and Gong, 2018). The lengthening of the villi could promote the absorption of minerals, nutrients, and water; this has a positive association with fecal excretion and can improve constipation. The physiometabolic effects associated with the consumption of *A. salmiana* fructans on fecal excretion were also observed in a previous study (Castillo Andrade et al., 2018).

The intestinal mucosal epithelial cells are covered with membrane-bound mucins (MUC3 and MUC4), providing a barrier that protects against enteropathogenic bacteria and viruses infiltrating the inner mucosa layer (Paturi et al., 2015).

Disruption of the mucosa layer and the reactive changes in the shape and size of cells, however, were observed in the doses of 15 and 20%. We hypothesize that these doses have an adverse effect associated with the deregulation of intestinal homeostasis conducted by the excessive production of organic acids by intestinal bacteria. This finding has been reported in ruminants: when their diet is high in grain, subacute ruminal acidosis occurs because of an increase of SCFAs, which leads to endotoxin production causing the severe damage of rumen epithelium (Tao et al., 2014). The alterations in the gut structure, such as morphological changes of intestinal glands and architecture, and modifications at the order/phylum level (dysbiosis) have an effect on gut homeostasis, microbiota functionality, and the health status of the colon. Gastric atrophy could cause infection by several pathogens, such as the Enterobacteriaceae group (Pédron and Sansonetti, 2008). The disruption of the mucosa layer in these doses creates an imbalance in mucus defensive mechanisms and undoubtedly represents a persistent risk of microbial invasion.

The disruption of the mucosa immune barrier favors the colonization and growth of pathogens within the gastrointestinal tract, which then invades the host tissue and causes gut diseases (Al-Sheraji et al., 2013).

In cells of rats fed with 20% of fructans, the presence of macrophages and CD4 T lymphocytes was observed because of the activation of the inflammatory response that begins to repair the mucosa layer in both tissues. If it is perpetuated for a longer time with damage, it will cause an alteration and cicatrization demonstrated by the increase in collagen production, as was observed in the tissue of the animals fed with 20% fructans. It was observed that cellular metabolites, such as dendritic cells and subsequent mucin production, occur after 15 days of the inflammation process (Oros-Ovalle personal communication). These same results were observed by Moreno-Vilet et al. (2014), who investigated the *in vitro* activity of *A. salmiana* fructans (2%) as an immune system activator, demonstrating that these types of fructans are involved in the activation and selective differentiation of cells of the immune system through interactions with bacterial lactic acid.

It could be interesting to evaluate, however, if the damage caused by high doses of fructans (15–20%) at 35 days of treatments is reversible.

The literature indicates that the consumption of a fiber-rich diet is associated with a decreased risk of gastrointestinal diseases such as colon cancer; however, until now no definitive statements can be made about the mode of action of specific types or the amounts of dietary fiber. There is a higher incidence of diverticular disease in old age, which is currently believed to be associated with localized inflammation, and this is exacerbated by a low fiber diet (Comparato et al., 2007). This research provides the first evidence of a positive correlation *in situ* between the consumption of *A. salmiana* fructans and gut epithelium health. Our results suggest that a dose not more than 12.5% can be considered the best dose for stimulating higher mucus production without causing cytotoxicity and improving the gastroprotective function of the colonic epithelium. These data are consistent with prebiotic effects findings in our previous study (Castillo Andrade et al., 2019).

The observation through the SEM of the cecum and proximal colon concurs with the prebiotic effect of *A. salmiana* fructans reported by Castillo Andrade et al. (2019), and they are associated with the results of the histological analysis of this current study.

Within the prebiotic properties attributed to fructans is the ability to alter the gut pathogenesis and to act as blocking factors, dislodging the adherent pathogen of the mucosa layer (Al-Sheraji et al., 2013). In the administration of a dose of 12.5%, it is possible to observe numerous cylindrical villi and an increase of bacterial population attached to the rat colon epithelium. In the case of a 15% dose, a decrease in intestinal microbiota was observed. This decrease can negatively affect the wellbeing of the host by causing metabolic disorders, chronic inflammation, or the sensitization of the host to infectious diseases (Cani et al., 2009; Spiller and Garsed, 2009). Therefore, the intake of fructans at the 15% dose is not recommended.

The present study also indicated a significant decrease in the concentration of TNF-α in the serum of the intervention group fed with 10 and 12.5% compared with the other intervention groups and the control after 7 days of treatment. These levels remained constant until 35 days of treatment. Our finding suggested that the major change of TNF-α levels occurs in the first 7 days, which can be attributed directly to the reduction of inflammation in the epithelium of the cecum and proximal colon associated with the consumption of fructans of *A. salmiana*. Some studies have demonstrated that the level of pro-inflammatory cytokine TNF-α was decreased in response to supplementation with prebiotic and probiotic strains, such as *Lactobacillus* and/or *Bifidobacterium* (Fukoshima et al., 2007; Schiffrin et al., 2009; Zaharuddin et al., 2019), which has been associated with reduced inflammation.

Despite the damages and changes in the structure of the cecum and proximal colon epithelium observed previously in the 15 and 20% doses, the anti-inflammatory capacity of the fructans of *A. salmiana* through decreasing the concentration of TNF-α was corroborated. This suggests the possible participation of inhibitory mechanisms at the level of the transcription factor NFκB and the activation of the healing process. We considered that the administration of 12.5% of fructans in the human diet could improve human gut health, because diets with high prebiotic contents include beneficial to host nutrition, promotes beneficial microbiota, and decrease chronic diseases.

## Conclusion

This study provides several lines of evidence to demonstrate that administering *A. salmiana* fructans in a maximum dose of 12.5% to the diet provides beneficial effects in the health of the colonic epithelium of Wistar rats. The increase in mucus secretion in doses of 10 and 12.5% allowed the increase and adhesion of beneficial bacteria that contribute to the prebiotic effect of the fructans and the suppression of epithelium inflammation, which is shown through the decreased expression of the pro-inflammatory TNF-α cytokine. For doses higher than 12.5%, the inflammatory process was observed, however. The results provide a solid basis for future clinical trials of the oral administration of *A. salmiana* fructans as functional ingredients for the primary prevention of intestinal diseases.

## Data Availability Statement

The original contributions presented in the study are included in the article, further inquiries can be directed to the corresponding author/s.

## Ethics Statement

The animal study was reviewed and approved by the Local Ethics Committee for Animal Research of the Faculty of Chemical Science under registration number CONBIO TICA24CEI00820131212. Written informed consent for participation was not obtained from the owners, because the rats were donated by the Laboratory Animal Center of the Faculty of Medicine.

## Author Contributions

AC, AG, and EG designed the research plan. CO contributed with his expertise on histopathological analyses and discussed these results. AC and CR performed the experiments. AC and AG analyzed and discussed the results and wrote the manuscript. MR revised and corrected the English version. All authors revised the manuscript, contributed to the article, and approved the submitted version.

## Conflict of Interest

The authors declare that the research was conducted in the absence of any commercial or financial relationships that could be construed as a potential conflict of interest.

## Publisher’s Note

All claims expressed in this article are solely those of the authors and do not necessarily represent those of their affiliated organizations, or those of the publisher, the editors and the reviewers. Any product that may be evaluated in this article, or claim that may be made by its manufacturer, is not guaranteed or endorsed by the publisher.

## References

[B1] Aguirre-Rivera J. R. Charcas-Salazar H. Flores-Flores J. L. (2001). *El Maguey Mezcalero Potosino.* San Luis Potosí, MX: Universidad Autónoma de San Luis Potosí, 78.

[B2] Al-Sheraji S. H. Ismail A. Manap M. Y. Mustafa S. Yusof R. M. Hassana F. A. (2013). Prebiotics as functional foods: a review. *J. Funct. Foods* 5 1542–1553. 10.1016/j.jff.2013.08.009

[B3] Barnali S. Ghosh S. (2014). Gastrointestinal microbiota in *Oreochromis mossambicus* (Peters) and *Oreochromis niloticus* (Linnaeus): scanning electron microscopy and microbiological study. *Int. J. Fish. Aquat. Stud.* 2 78–88.

[B4] Bedford A. Gong J. (2018). Implications of butyrate and its derivatives for gut health and animal production. *Anim. Nutr.* 4 151–159. 10.1016/j.aninu.2017.08.010 30140754 PMC6104520

[B5] Biagi E. Candela M. Fairweather-Tait S. Franceschi C. Brigidi P. (2012). Ageing of the human metaorganism: the microbial counterpart. *Age* 34 247–267. 10.1007/s11357-011-9217-5 21347607 PMC3260362

[B6] Brownlee I. A. Havler M. E. Dettmar P. W. Allen A. Pearson J. P. (2003). Colonic mucus: secretion and turnover in relation to dietary fibre intake. *Proc. Nutr. Soc.* 62 245–249. 10.1079/pns2003206 12756974

[B7] Canani B. R. Di Costanzo M. Leone L. (2012). The epigenetic effects of butyrate: potential therapeutic implications for clinical practice. *Clin. Epigenet.* 4:4. 10.1186/1868-7083-4-4 22414433 PMC3312834

[B8] Cani P. D. Possemiers S. Van de Wiele T. Guiot Y. Everard A. Rottier O. (2009). Changes in gut microbiota control inflammation in obese mice through a mechanism involving GLP-2-driven improvement of gut permeability. *Gut* 58 1091–1103. 10.1136/gut.2008.165886 19240062 PMC2702831

[B9] Castillo Andrade A. I. C Rivera Bautista C. Godínez-Hernández C. Ruiz-Cabrera M. A. Fuentes-Ahumada C. García-Chávez E. (2018). Physiometabolic effects of *Agave salmiana* fructans evaluated in Wistar rats. *Int. J. Biol. Macromol.* 108 1300–1309. 10.1016/j.ijbiomac.2017.11.043 29138000

[B10] Castillo Andrade A. I. C Rivera Bautista C. Ruiz Cabrera M. A. Soria Guerra R. E. Fuentes Ahumada C. García Chávez E. (2019). Agave fructans as gut health promoters: prebiotic activity and inflammatory response in Wistar healthy rats. *Int. J. Biol. Macromol.* 136 785–795. 10.1016/j.ijbiomac.2019.06.045 31189087

[B11] Comparato G. Pilotto A. Franzè A. Franceschi M. Di Mario F. (2007). Diverticular disease in the elderly. *Dig. Dis.* 25 151–159. 17468551 10.1159/000099480

[B12] Daly K. Cuff M. A. Fung F. Shirazi-Beechey S. P. (2005). The importance of colonic butyrate transport to the regulation of genes associated with colonic tissue homoeostasis. *Biochem. Soc. Trans.* 33 733–735. 10.1042/bst0330733 16042588

[B13] Fukoshima Y. Miyaguchi S. Yamano T. Kaburagi T. Iino H. Ushida K. (2007). Improvement of nutritional status and incidence of infections in hospitalised, enterally fed elderly by feeding of fermented milk containing probiotic *Lactobacillus johnsonii* La1 (NCC533). *Br. J. Nutr.* 98 969–977. 10.1017/S0007114507764723 17617944

[B14] Gibson G. R. Hutkins R. Sanders M. E. Prescott S. L. Reimer R. A. Salminen S. J. (2017). Expert consensus document: The International Scientific Association for Probiotics and Prebiotics (ISAPP) consensus statement on the definition and scope of prebiotics. *Nat. Rev. Gastroenterol. Hepatol.* 14 491–500. 10.1038/nrgastro.2017.75 28611480

[B15] Jasso-Padilla I. Juárez-Flores B. Álvarez-Fuentes G. De la Cruz-Martínez A. González-Ramírez J. Moscosa-Santillán M. (2016). Effect of prebiotics of *Agave salmiana* fed to healthy Wistar rats. *J. Sci. Food Agric.* 97 556–563. 10.1002/jsfa.7764 27097820

[B16] Laparra J. M. Sanz Y. (2010). Interactions of gut microbiota with functional food components and nutraceuticals. *Pharm. Res.* 61 219–225. 10.1016/j.phrs.2009.11.001 19914380

[B17] Martinez-Gutierrez F. Ratering S. Juárez-Flores B. Godinez-Hernandez C. Rita Geissler-Plaum R. Prell F. (2017). Potential use of *Agave salmiana* as a prebiotic that stimulates the growth of probiotic bacteria. *LWT Food Sci. Tech.* 84 151–159. 10.1016/j.lwt.2017.05.044

[B18] Martin-Pérez S. (2000). Técnica (MARPER) para la inyección intravenosa y obtención de sangre de la vena caudal de la rata. *Rev. Hispan. Cien. Anim. Lab.* 5:18.

[B19] Moreno-Vilet L. García-Hernández M. H. Delgado-Portales R. E. Corral-Fernández N. M. Cortez-Espinoza N. Ruiz-Cabrera M. A. (2014). In vitro assessment of agave fructans (*Agave salmiana*) as prebiotics and immune system activators. *Int. J. Biol. Macromol.* 63 181–187. 10.1016/j.ijbiomac.2013.10.039 24211431

[B20] Paßlack N. Vahjen W. Zentek J. (2015). Dietary inulin affects the intestinal microbiota in sows and their suckling piglets. *BMC Vet. Res.* 11:51. 10.1186/s12917-015-0351-7 25889573 PMC4352559

[B21] Paturi G. Butts C. A. Bentley-Hewitt K. L. Hedderley D. H. Stoklosinski H. Ansell J. (2015). Differential effects of probiotics prebiotics, and synbiotics on gut microbiota and gene expression in rats. *J. Funct. Foods* 13 204–213. 10.1016/j.jff.2014.12.034

[B22] Pédron T. Sansonetti P. (2008). Commensals, bacterial pathogens and intestinal inflammation: an intriguing ménage à trois. *Cell Host Microbe* 3 344–347. 10.1016/j.chom.2008.05.010 18541210

[B23] Perrin P. Pierre F. Patry Y. Champ M. Berreur M. Pradal G. (2001). Only fibres promoting a stable butyrate producing colonic ecosystem decrease the rate of aberrant crypt foci in rats. *Gut* 48 53–61. 10.1136/gut.48.1.53 11115823 PMC1728184

[B24] Ramnani P. Costabile A. Bustillo A. G. R. Gibson G. R. (2015). A randomised, double-blind, cross-over study investigating the prebiotic effect of agave fructans in healthy human subjects. *J. Nutr. Sci.* 4:e10. 10.1017/jns.2014.68 26090092 PMC4463010

[B25] Rogler G. Andus T. (1998). Cytokines in inflammatory bowel disease. *World. J. Surg.* 22 382–389. 9523521 10.1007/s002689900401

[B26] Schaafsma G. Slavin J. L. (2015). Significance of inulin fructans in the human diet. *Compr. Rev. Food Sci. Food Saf.* 14 37–47. 10.1111/1541-4337.12119 33401810

[B27] Schiffrin E. J. Parlesak A. Bode C. Bode J. C. Van’t Hof M. A. Grathwohl D. (2009). Probiotic yogurt in the elderly with intestinal bacterial overgrowth: endotoxaemia and innate immune functions. *Br. J. Nutr.* 101 961–966. 10.1017/s0007114508055591 19353762

[B28] Spiller R. Garsed K. (2009). Infection, inflammation and the irritable bowel syndrome. *Dig. Liver Dis.* 12 844–849. 10.1016/j.dld.2009.07.007 19716778

[B29] Tao S. Duanmu Y. Dong H. Tian J. Ni Y. Zhao R. (2014). A high-concentrate diet induced colonic epithelial barrier disruption is associated with the activating of cell apoptosis in lactating goats. *BMC Vet. Res.* 10:235. 10.1186/s12917-014-0235-2 25256013 PMC4180839

[B30] Van de Wiele T. Boon N. Possemiers S. Jacobs H. Verstraete W. (2004). Prebiotic effects of chicory inulin in the simulator of the human intestinal microbial ecosystem. *FEMS Microbiol. Ecol.* 51 143–153. 10.1016/j.femsec.2004.07.014 16329863

[B31] Zaharuddin L. Mokhtar N. M. Nawawi K. N. M. Ali R. A. R. (2019). A randomized double-blind placebo controlled trial of probiotics in post-surgical colorectal cancer. *BMC Gastroenterol.* 19:131. 10.1186/s12876-019-1047-4 31340751 PMC6657028

